# Knowledge distillation of multi-scale dense prediction transformer for self-supervised depth estimation

**DOI:** 10.1038/s41598-023-46178-w

**Published:** 2023-11-02

**Authors:** Jimin Song, Sang Jun Lee

**Affiliations:** 1 Division of Electronic Engineering, Jeonbuk National University, 567 Baekje-daero Deokjin-gu, Jeonju, 54896 Korea; 2 Future Semiconductor Convergence Technology Research Center, Division of Electronic Engineering, Jeonbuk National University, 567 Baekje-daero Deokjin-gu, Jeonju, 54896 Korea

**Keywords:** Computer science, Electrical and electronic engineering

## Abstract

Depth estimation is an inverse projection problem that estimates pixel-level distances from a single image. Although, supervised methods have shown promising results, it has intrinsic limitations in requiring ground truth depth from an external sensor. On the other hand, self-supervised depth estimation relieves the burden for collecting calibrated training data, while there is still a large performance gap between supervised and self-supervised methods. The objective of this study is to reduce the performance gap between the supervised and self-supervised approaches. The loss function of previous self-supervised methods is mainly based on a photometric error, which is indirectly computed from synthesized images using depth and pose estimates. In this paper, we argue that direct depth cue is more effective to train a depth estimation network. To obtain the direct depth cue, we employed a knowledge distillation technique, which is a teacher-student learning framework. The teacher network was trained in a self-supervised manner based on a photometric error, and its predictions were utilized to train a student network. We constructed a multi-scale dense prediction transformer with Monte Carlo dropout, and multi-scale distillation loss was proposed to train the student network based on the ensemble of stochastic estimates. Experiments were conducted on the KITTI and Make3D datasets, and our proposed method achieved the state-of-the-art accuracy in self-supervised depth estimation. Our code is publicly available at https://github.com/ji-min-song/KD-of-MS-DPT.

## Introduction

Recently, deep learning based visual perception algorithms have been utilized in many industry problems. Among them, depth estimation has received much attention to acquire 3D information from a monocular camera. The objective of depth estimation is to predict pixel-level distances from the camera coordinate, and it has been applied to augmented reality^1^, robot navigation^2^, and autonomous driving^3^. However, due to the scale-ambiguity in the inverse projection problem, it has many additional challenges compared to other computer vision tasks which infer 2D semantic information. In this paper, we propose a novel self-supervised learning pipeline to improve the accuracy of monocular depth estimation.

Depth estimation methods can be categorized into supervised and unsupervised approaches. Supervised methods require external sensor data, and 3D LiDAR points are widely utilized to generate precise supervisory signals. However, it requires expensive LiDAR sensors and external calibration work to train a depth estimation model. On the other hand, unsupervised approach can be a promising alternative to reduce the burden for collecting calibrated ground truth data. The unsupervised approach is also referred to as self-supervised depth estimation because the loss can be computed from the information obtained by the camera itself. The loss function of most unsupervised methods is mainly based on a photometric error, and it can be computed from stereo pairs and/or monocular sequential images. To compute the photometric error, an image is synthesized at a different viewpoint, and therefore, most self-supervised approaches require estimating both pixel-level distances and relative camera pose, simultaneously. Due to the complexity of the self-supervised approach, there is a large performance gap between the supervised and self-supervised methods.

**Figure 1:**
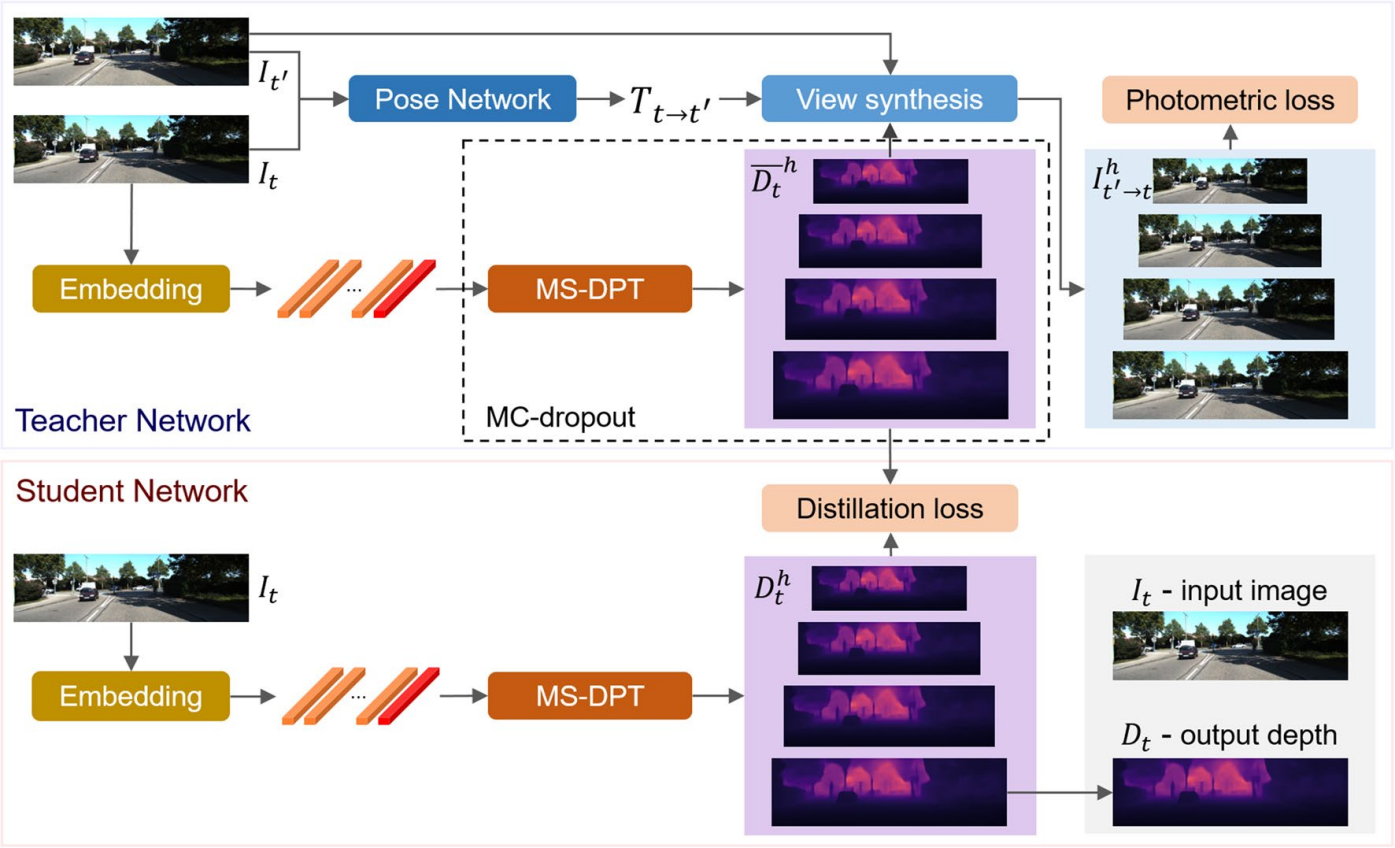
Overview of the proposed knowledge distillation framework. The teacher and student networks are composed of the multi-scale dense prediction transformer (MS-DPT). The teacher network is trained based on a photometric error at multiple scales, and the teacher network generates Monte Carlo samples for multi-scale depth maps. The ensemble of the multi-scale depth estimates at scale *h* is denoted by ${\overline{D_t}}^h$, and it is distilled to train the student network based on the proposed distillation loss.

The objective of this paper is to reduce the performance gap between the supervised and self-supervised methods. In this paper, we argue that direct depth cue is more effective to train a depth estimation module compared to the previous approach that is mainly based on a photometric error. The previous approach requires the training of depth and pose networks simultaneously, increasing the complexity of the learning process. On the other hand, the proposed method separates the procedure for obtaining the direct depth cue and the process for training a depth network. We adopted a knowledge distillation technique to generate the direct depth cue and employ it for the training of the depth network. In our proposed method, the teacher and student networks were constructed based on a transformer architecture which has shown the state-of-the-art performance in many computer vision tasks^5–7^. We modified the architecture of the dense prediction transformer (DPT)^8^ and added multi-scale prediction heads to produce depth maps at multiple scales. We refer to the proposed transformer architecture as Multi-Scale Dense Prediction Transformer (MS-DPT). To improve the reliability of the direct depth cue, we employed Monte Carlo dropout (MC-dropout)^9^ and generated an ensemble of Monte Carlo (MC) estimates. Finally, multi-scale distillation loss was proposed to distill the knowledge in the multi-scale version of the depth map ensemble. Figure 1 presents the overview of the proposed knowledge distillation framework. Experiments were conducted on the KITTI^10^ and Make3D^11^ datasets, and our proposed method achieved the state-of-the-art accuracy on most fundamental metrics in self-supervised depth estimation.

## Related work

### Monocular depth estimation

Supervised depth estimation has been studied in various aspects including network architectures and loss functions. Eigen et al.^12^ proposed a scale-invariant loss which has been utilized as a fundamental metric for evaluating depth estimation techniques. Fu et al.^13^ proposed DORN which integrates an ordinal regression layer to estimate depth maps. By introducing a spacing-increasing discretization strategy, DORN reduced the absolute relative error especially at close distances. Li et al.^14^ proposed another ordinal regression method called BinsFormer, and it generated adaptive bins based on a transformer architecture. Ranftl et al.^8^ demonstrated the effectiveness of pretraining on a large-scale dataset for improving depth estimation performance. They proposed a vision transformer architecture for dense prediction tasks and achieved the state-of-the-art performance on the KITTI dataset by incorporating pretrained parameters trained on multiple datasets.

Recently, self-supervised depth estimation methods have received a lot of attention due to its intrinsic benefit of not requiring ground truth data. Godard et al.^15^ proposed Monodepth2 which is a self-supervised learning pipeline. By introducing a novel photometric error and auto-masking technique, it addressed the problem of depth predictions at occluded regions and moving objects. Yan et al.^16^ proposed CADepth-Net which contains two different attention modules for emphasizing the structure and details. Jiang et al.^17^ introduced a modified photometric error between a reconstructed image and the corresponding adjacent images. Chawla et al.^18^ proposed a depth estimation framework that employs a global positioning system as an additional supervision for pose estimation. Guizilini et al.^19^ and Lu et al.^20^ introduced novel self-supervised learning methods that jointly learn depth and optical flow. Varma et al.^21^ proposed MT-SfMLearner that employs the DPT^8^ architecture for both depth and pose networks and utilized camera intrinsic parameters as an intermediate supervision for the training of the pose network. In this work, we propose a novel self-supervised learning framework based on knowledge distillation of a transformer architecture.

### Knowledge distillation for depth estimation

Knowledge distillation of deep neural networks was firstly proposed by Hinton et al.^4^ to effectively reduce the model size. Hinton et al. demonstrated that probabilistic responses of a large teacher network is beneficial to train a smaller student network. In the teacher-student learning framework, various loss functions have been proposed to distill the knowledge in intermediate feature maps^22^ and relationships between data samples^23^. Recently, knowledge distillation has been utilized to improve the accuracy of a teacher network^24^, and it has been successfully applied to many computer vision tasks including object detection^25^, segmentation^26^, and domain adaptation^27^.

Knowledge distillation has been applied to address the problem of monocular depth estimation. Wang et al.^28^ proposed a novel knowledge distillation method for depth estimation in edge devices. Song et al.^29^ proposed a method to selectively distill the knowledge in pixel-level distances from a pre-trained stereo depth network to a monocular depth network. Pilzer et al.^30^ introduced a cyclic pipeline that utilizes reconstructed right images computed from reconstructed left images and predicted right depth maps. In this paper, we propose a novel knowledge distillation framework for handling multi-scale depth maps generated from our teacher network.

## Proposed method

This section presents the proposed knowledge distillation framework to address the problem of self-supervised depth estimation. We first explain the architecture of the proposed deep learning model called MS-DPT, which is employed in both teacher and student networks. Subsequently, we present the process for distilling the knowledge in the multi-scale predictions of the teacher network to train the student network.

### Network architecture

**Figure 2:**
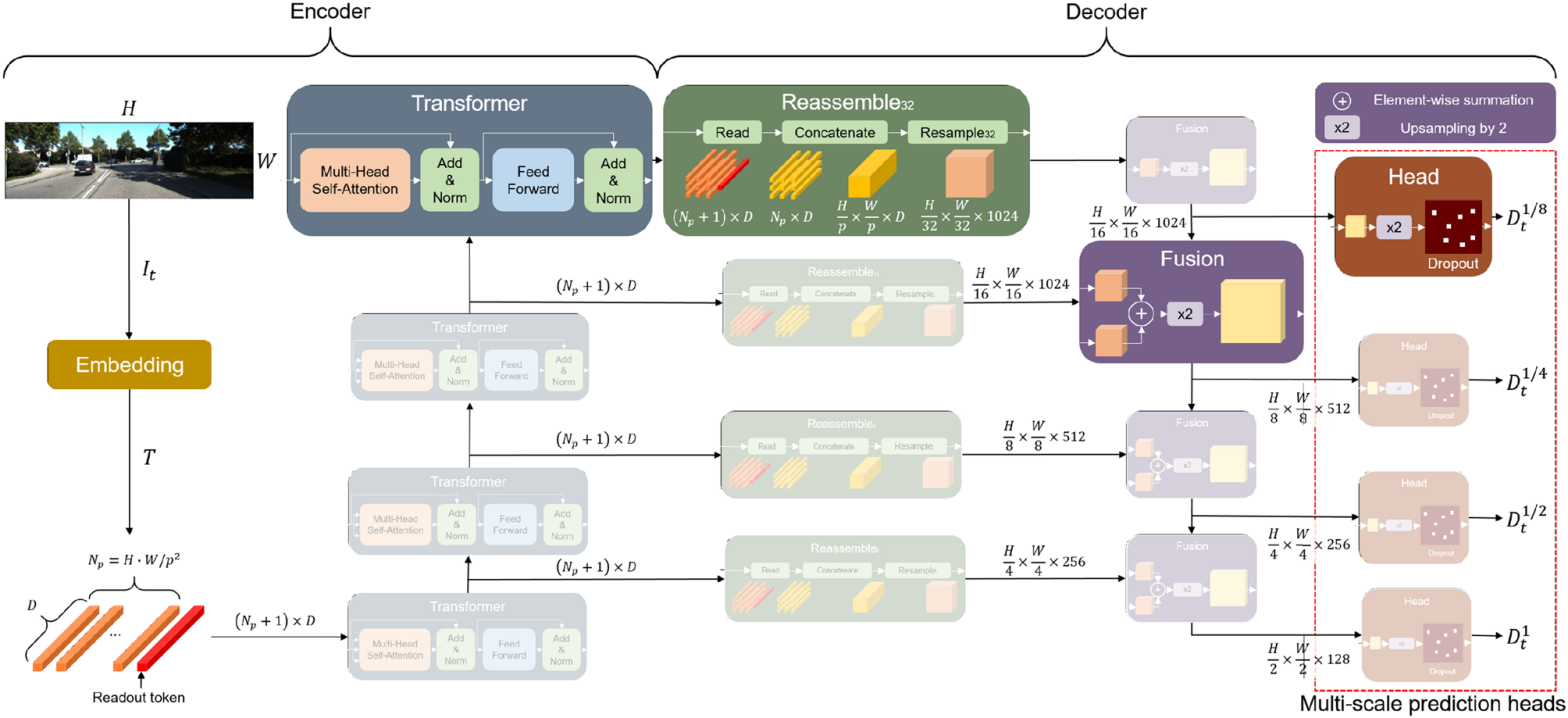
Architecture of the proposed depth estimation network. The MS-DPT model consists of the transformer encoder in the DPT model^8^ and multi-scale prediction heads. The multi-scale depth maps are fed into the distillation loss to train the student network.

Our proposed model was designed by modifying the DPT^8^ architecture. The DPT model was proposed for supervised depth estimation and segmentation tasks, and it consists of transformer modules. We added multi-scale prediction heads to extract feature maps at multiple scales, and Fig. 2 presents the details of our depth estimation network. The encoder part consists of an embedding layer and transformer blocks. The embedding layer converts an input image $I \in R^{H \times W \times 3}$ into a set of tokens $T \in R^{(N_p + 1)\times D}$, where *H* and *W* are the height and width of the input image. In the embedding layer, the input image is divided into $p \times p$ non-overlapping patches, and these patches are converted into $N_p = {H \cdot W} / p^2$ tokens in which each token is a $D$-dimension vector. It was implemented by a $p \times p$ convolution with the stride *p*, and a learnable vector named readout token is concatenated to the tokens. A transformer block consists of layer normalization, multi-head self-attention, residual connection, and multi-layer perceptron (MLP) layer. Identical to the DPT-Large model, the hidden size *D* and the number of transformer blocks *L* were set to 1024 and 4, respectively, to utilize the pretrained parameters trained on the MIX 6 dataset^8^. To integrate feature representations, the outputs of the transformer blocks are fed into the decoder part consisting of reassemble blocks and multi-scale prediction heads.

The decoder part consists of reassemble blocks, fusion blocks, and head blocks. A reassemble block converts a token set into an image-like feature map, a fusion block doubles the spatial size of the feature map and a head block performs the downstream task. There are three operations in each reassemble block: $Read$, $Concatenate$, and $Resample_s$ operations. In the $Read$ operation, a readout token is concatenated to each token in token set $T$, and it is projected to ${\mathbb {R}}^{{N_p} \times D}$ through a MLP layer. The $Concatenate$ operation rearranges the projected token set into an image-like features map $F' \in R^{{\frac{H}{p}} \times {{\frac{W}{p}}} \times D}$. The $Resample_s$ operation converts the channel and the spatial size of $F'$ into $F \in R^{{\frac{H}{s}} \times {{\frac{W}{s}}} \times {\hat{D}}}$ by utilizing $1 \times 1$ convolution and bilinear interpolation. In each $Resample_s$ operation, the channel size $\hat{D} \in \{256, 512, 1024, 1024\}$ decreases as the spatial size $s \in \{4, 8, 16, 32\}$ increases. The output of the reassemble block is added to the output of the previous fusion block, and the spatial size is doubled by the residual convolution unit proposed in RefineNet^31^.

Different to the original DPT^8^, our MS-DPT contains multi-scale prediction heads to generate multi-scale depth maps as presented in Figure 2. A scaled depth map at scale $h \in \{1, 1/2, 1/4, 1/8\}$ is generated from the output of the fusion block, which is fed into the head block. A head block is composed of $3 \times 3$ and $1 \times 1$ convolutions, and the feature maps are upsampled by a bilinear interpolation. By optimizing depth network with multi-scaled depth maps, the network can progressively learn more meaningful representations compared to when using a single inference result. The effectiveness of the multi-scale prediction heads is analyzed in experimental results.

### Teacher network

In our knowledge distillation framework, a MS-DPT model is trained as a teacher network by following the self-supervised learning pipeline proposed in Monodepth2^15^. Training the teacher network requires stereo pairs and/or monocular sequential images, and we denote a pair of adjacent images as $I_t$ and $I_{t'}$ in the following description. Adjacent images $I_t$ and $I_{t'}$ are fed into a pose network to infer the relative pose $T_{t \rightarrow t'}$ from the viewpoint of $I_t$ to the viewpoint of $I_{t'}$. The pose network was constructed based on the architecture of ResNet^32^, and it infers the Euclidean transformation between the two viewpoints. Simultaneously, the teacher network infers the multi-scale depth map, and $D_t^h$ denotes the predicted depth maps at scale *h* for the input image $I_t$. As presented in Fig. 2, we utilized four different scales, and the scale factor is denoted by $h \in \{1, 1/2, 1/4, 1/8\}$. For each scale *h*, the reconstructed image $I_{t' \rightarrow t}^h$ can be computed as follows:

1 $$\begin{aligned} I_{t' \rightarrow t}^h = I_{t'}^h \Big \langle proj({D_t}^h, T_{t \rightarrow {t'}}^h, K^h) \Big \rangle , \end{aligned}$$

where $I_{t'}^h$, $T_{t \rightarrow t'}^h$, and $K^h$ are the scaled version of $I_t'$, $T_{t \rightarrow t'}$, and the intrinsic parameter *K*, respectively. In (1), $proj(\cdot )$ is the projection operation onto the image plane at the viewpoint of $I_t$, and $\langle \cdot \rangle$ is the differentiable sampling operation.

The photometric error between an input $I_t^h$ at scale *h* and the corresponding reconstructed image $I_{t' \rightarrow t}^h$ is computed based on the combination of the structural similarity index measure (SSIM)^33^ and L1-norm as follows:

2 $$\begin{aligned} \begin{aligned} pe(I_t^h, I_{t' \rightarrow t}^h) = \frac{\alpha }{2}(1-SSIM(I_t^h, I_{t' \rightarrow t}^h)) + (1-\alpha )\Vert I_t^h - I_{t' \rightarrow t}^h\Vert _1. \end{aligned} \end{aligned}$$

In experiments, the heuristic parameter $\alpha$ was set to 0.85. Following the previous work^15^, we compute the minimum photometric error among multiple adjacent images to handle occluded regions as (3).

3 $$\begin{aligned} L_p^h = \mathop {\textrm{min}}\limits _{t'} \, pe(I_t^h,I_{t' \rightarrow t}^h). \end{aligned}$$

In the monocular training setup, $I_{t'}$ is a temporally adjacent image of $I_t$, and $I_{t'} \in \{I_{t-1},I_{t+1}\}$. On the other hand, in the monocular-stereo setup, adjacent images also include the opposite stereo view. The photometric error assumes the situation of moving camera and static objects. To compute the photometric error only at pixels projected from static environments, we employ the auto-masking technique utilized in Monodepth2^15^ and compute the scaled mask $\mu ^h$ at scale *h* as (4).

4 $$\begin{aligned} \mu ^h = [ \mathop {\textrm{min}}\limits _{t'} \, pe(I_t^h,I_{t' \rightarrow t}^h) < \mathop {\textrm{min}}\limits _{t'} \, pe(I_t^h,I_{t'}^h)], \end{aligned}$$

where $[\cdot ]$ is the Iverson bracket. Furthermore, we employ the edge-aware smoothness loss $L_s^h$ which was proposed by Godard et al.^34^ to produce smooth depth maps when the corresponding input regions have similar color intensities. The edge-aware smoothness loss is computed as follows:

5 $$\begin{aligned} L_s^h = | \partial _x d_t^{*} |e^{-| \partial _x I_t^h |} + | \partial _y d_t^{*} |e^{-| \partial _y I_t^h |}, \end{aligned}$$

where $d^*_t=d_t/ \overline{d_t}$ is the mean-normalized inverse depth. Finally, the total loss for the training of the teacher network is defined as (7).

6 $$\begin{aligned} L = \underset{h}{\sum }\ h^2 (\mu ^h L_p^h + \beta L_s^h). \end{aligned}$$

In experiments, the heuristic parameter $\beta$ was set to 0.001.

In the proposed method, the accuracy of the teacher network directly affects the accuracy of the student network. To improve the accuracy of the teacher network, we utilized the pretrained parameter of DPT which was trained on the mixing dataset MIX 6^8^. Moreover, we employ MC-dropout^9^ to generate multiple stochastic depth estimates from the teacher network. At each scale *h*, the averaged depth map is computed from MC estimates, and it is utilized to distill the knowledge in the teacher network to the student network. Although the sampling of multiple depth estimates causes computational complexity, it is conducted only in the training phase. The effectiveness of the multi-scale prediction head in MS-DPT and the ensemble of MC estimates is analyzed in experimental results.

### Student network

For the training of the student network, multiple MC estimates are inferred by the teacher network. The *i*-th MC estimate for the depth prediction at scale *h* is denoted by $D_{t,i}^h$. The stochastic samples of the multi-scale depth maps are averaged to improve the reliability of the predicted depth map at each scale. The averaged depth map at scale *h* is denoted by $\overline{D_t}^h$, and it can be computed as (7).

7 $$\begin{aligned} \overline{D_t}^h = \frac{1}{M} \underset{i=1}{\overset{M}{\sum }} D_{t,i}^h \end{aligned}$$

where *M* is the number of MC estimates.

To distill the knowledge in the multi-scale averaged depth map $\overline{D_t}^h$, we propose the following multi-scale distillation loss:

8 $$\begin{aligned} L_{distill} = \underset{h}{\sum }\ h^2 \sqrt{L_D^h}, \end{aligned}$$

where $L_D^h$ is the loss between $\overline{D_t}^h$ and $D_{t,S}^h$, $\overline{D_t}^h$ is the averaged depth estimate of the teacher network, and $D_{t,S}^h$ is the depth prediction of the student network at scale *h*. The loss between the depth estimates at scale *h* are measured based on the scale-invariant loss proposed by Eigen et al.^12^ as (9).

9 $$\begin{aligned} L_D^h = \frac{1}{n} \underset{i}{\sum }d^2_i - \frac{\lambda }{n^2} (\underset{i}{\sum }d_i)^2, \end{aligned}$$

where $d_i = \log {y_i^S} - \log {y_i^T}$, $\lambda = 0.85$, *n* is the number of valid pixels, and $y_i^T$ and $y_i^S$ are the depth estimates of the teacher and student networks at pixel *i*. We aim to transfer the knowledge of the teacher network by distilling the information contained in the multi-scale depth maps, and the effectiveness of the proposed method is demonstrated in experimental results.

## Experimental results

### Experiment setting and evaluation measures

Experiments were conducted on the hardware environment which includes AMD Ryzen9 5950X 16-Core, 32GB DDR4 RAM, and RTX 3090 GPU. The proposed algorithm was implemented by using Pytorch, and the code and trained model are available at https://github.com/ji-min-song/KD-of-MS-DPT. Transfer learning was conducted for the encoder part of MS-DPT by using the pretrained parameters of DPT which was trained on the MIX6 dataset. The use of the pretrained parameters significantly reduced the training time and improved the validation accuracy. The teacher network was trained by using a self-supervised loss function, which is mainly based on the photometric error. On the other hand, the student network was trained in a supervised manner by using a direct depth cue inferred by the teacher network. The training of the teacher and student networks took about 20 hours and 16 hours, respectively.

The performance of the proposed method was analyzed based on 6 error metrics and 3 accuracy metrics, which are utilized in evaluating depth estimation algorithms. Silog and AbsRel indicate the scale-invariant error and absolute relative error between estimated depth maps and the corresponding ground truth data. SqRel, RMSE, iRMSE and $\log _{10}$ indicate the square relative error, root mean squared error, root mean square of the inverse depth, and logarithmic error, respectively. Accuracy metrics compute the ratio of predicted pixels which have the relative error lower than a threshold value $\delta$. The relative error is computed by $\max (\hat{d}/d,d/\hat{d})$, where $\hat{d}$ and *d* are predicted and ground truth depth. Accuracy metrics are measured based on the three threshold values, and more details about the evaluation measures can be found in the previous work^12^.

### Experimental results on the KITTI benchmark dataset

This section presents experimental results of the proposed method on the KITTI dataset. The KITTI benchmark provides datasets and leaderboards for the development and evaluation of visual recognition algorithms for autonomous driving. The depth prediction task provides RGB images along with their corresponding LiDAR points and extrinsic parameters between camera and LiDAR sensors. We adopted the standard experiment setting for the depth prediction task proposed by Eigen et al.^12^. The Eigen split is composed of 39,810 monocular triplets for training, 4424 for validation, and 697 for evaluation.

**Figure 3:**
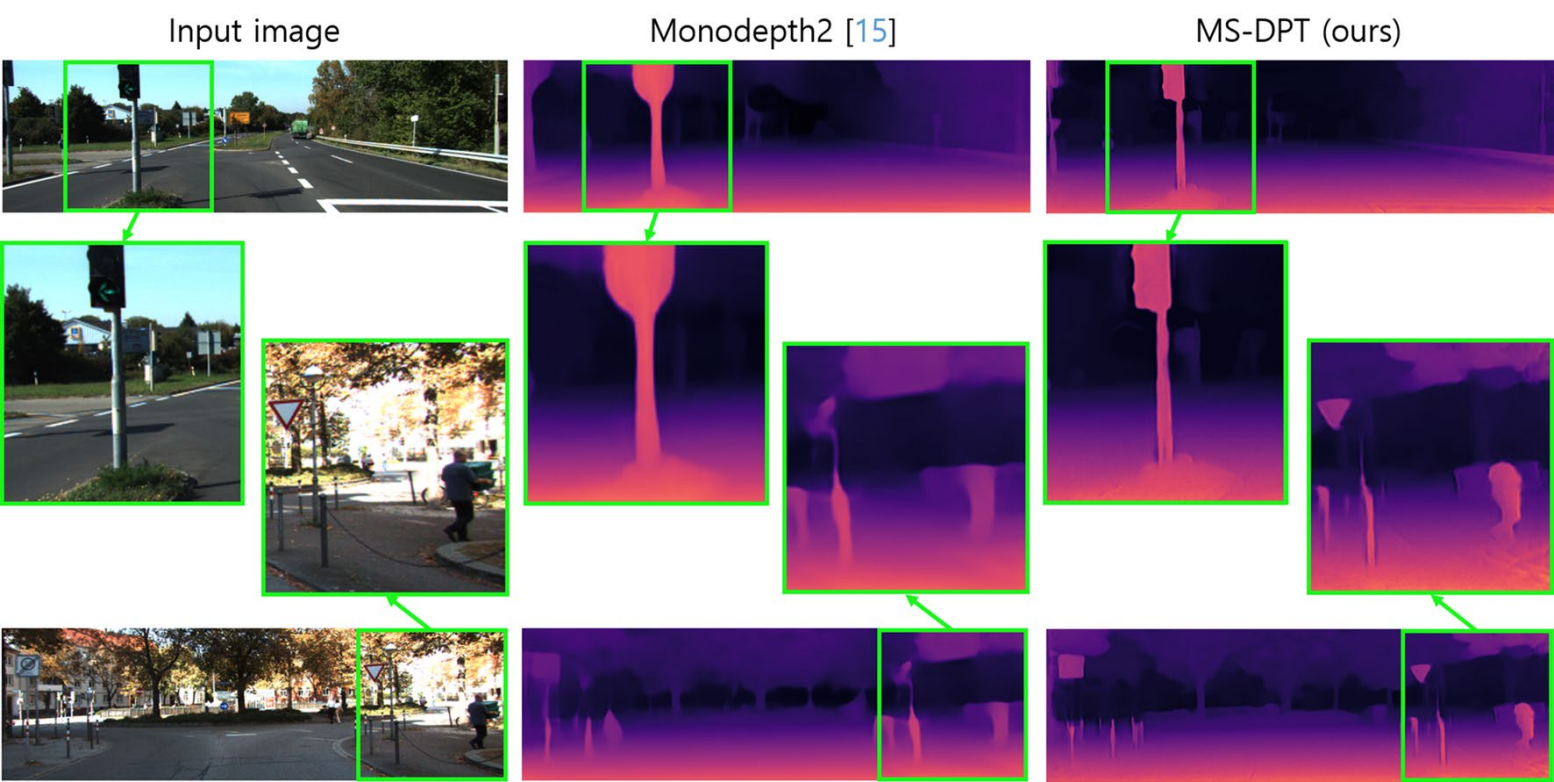
Qualitative results of the proposed algorithm on the KITTI dataset. Compared with Monodepth2, our proposed algorithm is superior to predict depth details for small objects such as traffic lights, traffic signs, and pedestrians. In the predicted depth maps, red and dark colors indicate near and far regions, respectively.

In the depth prediction task, there exist two types of ground truth data, referred to as the original and improved KITTI datasets. Raw LiDAR projections contain insufficient number of valid points to train a depth estimation algorithm. Therefore, the KITTI benchmark provides ground truth depth maps generated from 11 consecutive frames, and it is called the original KITTI dataset. On the other hand, Uhrig^35^ et al. proposed another type of ground truth data which were interpolated by using a convolutional neural network, and it is referred to as the improved KITTI dataset. The performance of supervised depth estimation algorithms was improved by utilizing the improved KITTI dataset. Although self-supervised methods do not employ ground truth data in the training process, some algorithms were evaluated on the original KITTI dataset while others were evaluated on the improved KITTI dataset. Therefore, we compared our proposed algorithm with previous self-supervised depth estimation methods on both the original and improved KITTI datasets. Table 1 presents the performance of the proposed method on the Eigen split, and it is compared with previous algorithms. Our proposed method achieved the state-of-the-art performance in terms of the SqRel and RMSE metrics on the original KITTI dataset. Although CADepth-Net^16^ showed lower AbsRel in the original KITTI dataset, our algorithm demonstrated lower Silog in the official KITTI benchmark as presented in Table 2, achieving higher ranking in the leaderboard. Moreover, our proposed method showed superior generalization performance with a significant margin compared to CADepth-Net^16^ as shown in Table 5. When evaluated on the improved KITTI dataset, the proposed method outperformed all previous methods in all performance metrics except the AbsRel error metric. Figure 3 presents qualitative results of the proposed algorithm on the KITTI dataset. As shown in Fig. 3, our proposed algorithm shows better results in predicting object boundaries compared to Monodepth2^15^.

**Table 1:**
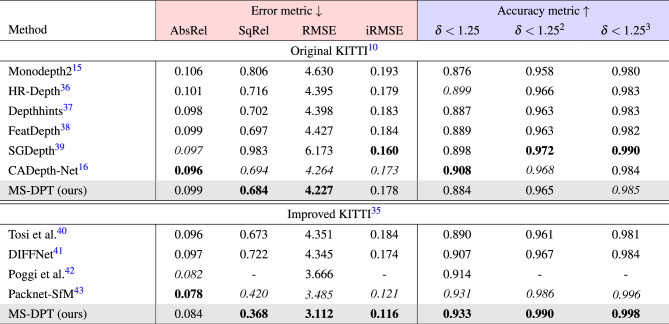
Quantitative results on the KITTI dataset.

For error metrics (red), the lower is the better, and for accuracy metrics (blue), the higher, is the better.

Significant values are in bold and italics.

**Figure 4:**
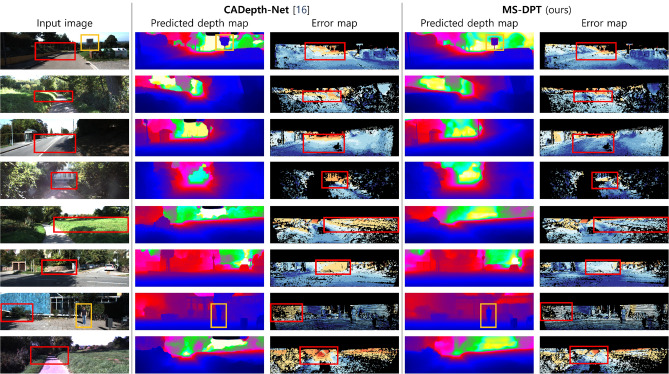
Qualitative results of the proposed algorithm on the KITTI test set. In the predicted depth maps, blue and red colors indicate near and far regions. In error maps, blue and red colors indicate small and large errors, respectively. The orange boxes in the predicted depth maps indicate depth details for the traffic sign and pedestrian, and the red boxes in the error maps highlight the small errors of the proposed method compared to the previous method. The original version of the predicted depth maps are available at the KITTI leaderboard.

The KITTI benchmark provides an online test server to compare the performance of depth estimation algorithms. In the test benchmark, previous algorithms are evaluated based on four error metrics, and their rankings are determined by Silog. Table 2 compares the performance of the proposed algorithm with previous methods. Our method achieved the state-of-the-art performance in self-supervised depth estimation, achieving the lowest error for Silog. In Fig. 4, the quality of depth maps predicted by our algorithm is compared with depth maps generated by CADepth-Net^16^, which achieved the second-best performance on the KITTI leaderboard. Figure 4 presents predicted depth maps and error maps produced by CADepth-Net^16^ and MS-DPT. The error maps were visualized in log scale, and blue and red colors indicate small and large errors, respectively. As shown in Fig. 4, MS-DPT is superior to predict depth details especially for thin-shaped objects such as traffic signs and road lights.

**Table 2:**
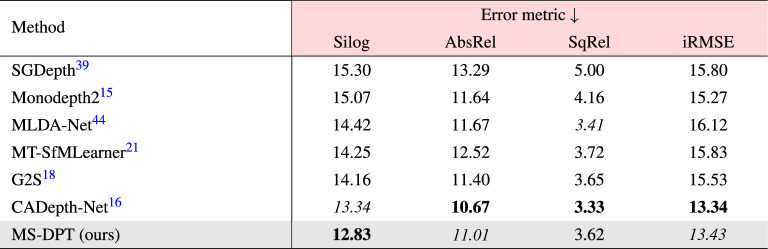
Experimental results of self-supervised depth estimation algorithms on the KITTI test set.

The ranking is determined based on the Silog error metric. The AbsRel error metric is expressed in percentage to facilitate comparison. The leaderboard can be available at https://www.cvlibs.net/datasets/kitti/eval_depth.php?benchmark=depth_prediction.

Significant values are in bold and italics.

### Analysis of the teacher and student networks

This section presents ablation studies to analyze the performance of the teacher and student networks. First, experiments were conducted to demonstrate the effectiveness of the MS-DPT architecture and the ensemble of its MC estimates. Table 3 compares the performance of our MS-DPT model with three previous depth estimation network architectures. These previous models were trained using the identical training pipeline to the self-supervised learning of our teacher network. Transfer learning was employed by utilizing the parameters of ResNet50 and DenseNet161 pretrained on the ImageNet-1k dataset for the Monodepth2 and BTS models, respectively. On the other hand, due to the different network architectures, the parameters of DPT pretrained on the MIX6 dataset were utilized for the DPT and MS-DPT models. As shown in Table 3, applying the multi-scale prediction head is beneficial in improving the depth accuracy, with the Silog error decreasing from 19.089 to 17.420. Furthermore, the performance of the teacher network was further improved by applying MC-dropout and the ensemble of MC estimates, with the Silog error decreasing from 17.420 to 17.158. In Table 3, the performance of the MS-DPT model without the ensemble of MC estimates is indicated by the dagger notation ($\dagger$). Our proposed model outperformed the previous methods in all metrics except the AbsRel error. Although our teacher model showed higher AbsRel compared to Monodepth2^15^, the following experimental results demonstrated that our proposed MS-DPT model is more effective for knowledge distillation.

**Table 3:**
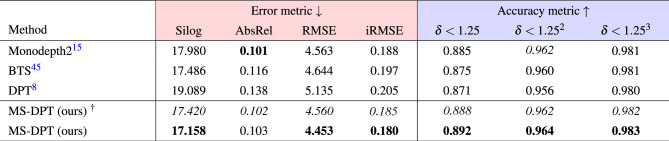
Performance analysis of the teacher model on the KITTI dataset.

The performance was compared with three network architectures for depth estimation. The dagger notation ($^{\dagger }$) indicates the performance of our teacher model without using the ensemble of MC estimates. For error metrics (red), the lower is the better, and for accuracy metrics (blue), the higher is the better.

Significant values are in bold and italics.

**Figure 5:**
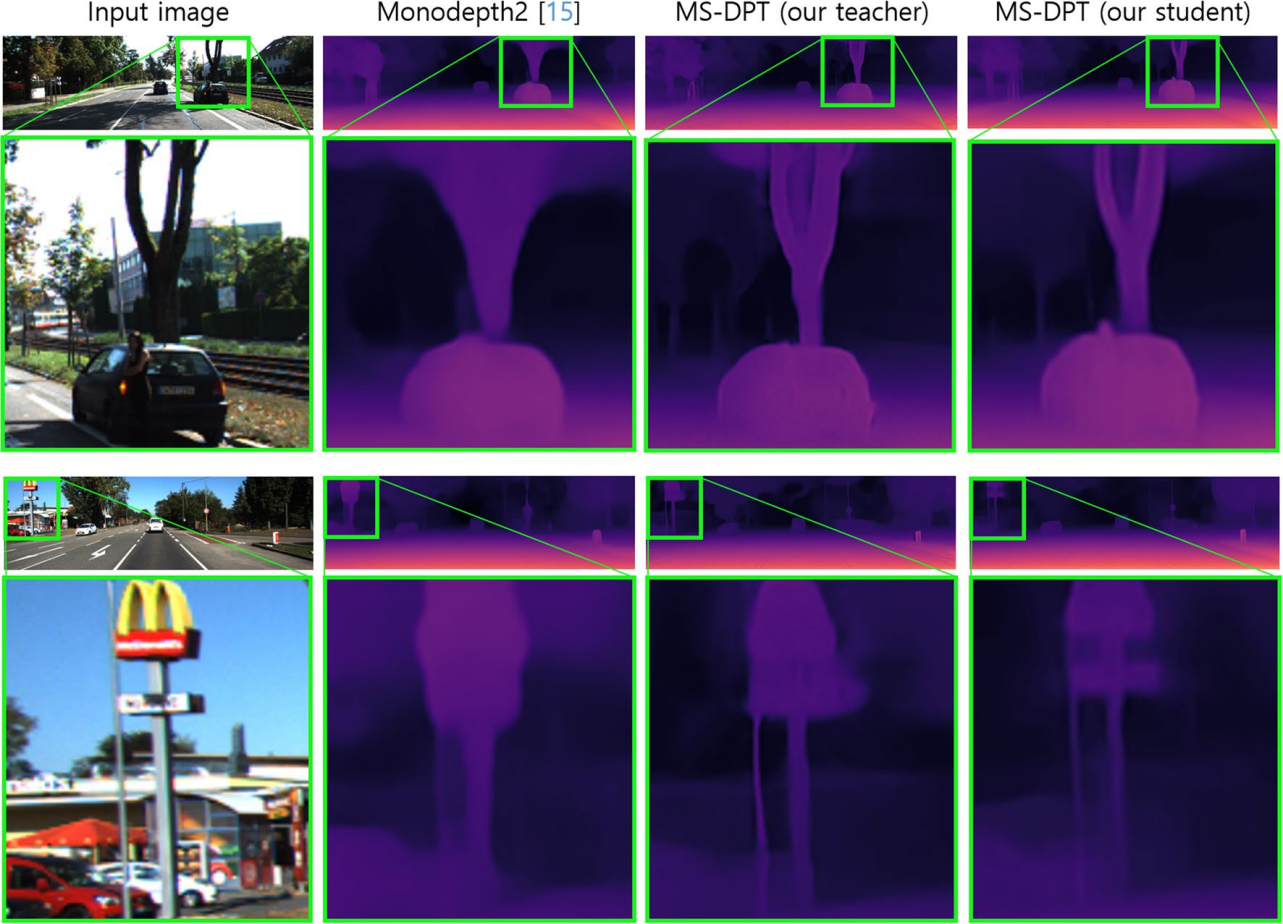
Qualitative comparison between the teacher and student models in the KITTI dataset.

We conducted experiments to demonstrate the effectiveness of knowledge distillation in self-supervised depth estimation, and experimental results are summarized in Table 4. The image resolution and self-supervision type affect to the performance of depth estimation. Therefore, we thoroughly conducted experiments with three self-supervision types including monocular, stereo, and monocular-stereo and two image resolution settings including 640$\times$192 and 1024$\times$320. In this experiment, we analyzed the proposed MS-DPT model comparing with the Monodepth2 model. When we trained teacher models with the architectures of Monodepth2 and MS-DPT, the Silog performance metrics were 17.951 and 17.158, respectively, for the input resolution of 1024$\times$320 and monocular-stereo supervision type. By distilling the knowledge of the two teacher networks of the Monodept2 and MS-DPT architectures, the Silog metrics were decreased from 17.951 to 16.970 and from 17.158 to 16.493, respectively. In Table 4, Monodepth2 $\rightarrow$ MS-DPT indicates that the teacher network with the architecture of Monodepth2 was distilled into the MS-DPT student model. Knowledge distillation consistently improves the depth estimation performance, and we obtained the best performance from the self-distillation of the MS-DPT model. Figure 5 presents the qualitative results of our teacher and student models. Object details were improved in the predicted depth maps of the student network compared to the teacher network. The depth cue generated by the teacher network contains the knowledge in the entire training dataset. We believe that the depth cue is more effective to train a depth network compared to the self-supervision computed from adjacent images.

**Table 4:**
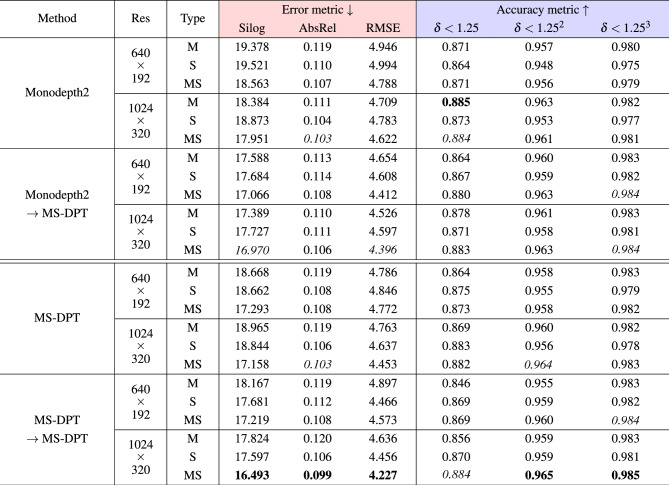
Effectiveness of knowledge distillation in self-supervised depth estimation.

In knowledge distillation, two teacher networks of Monodepth2 and MS-DPT architectures were analyzed for the training of the MS-DPT student model. Monodepth2 $\rightarrow$ MS-DPT indicates that the teacher network of Monodetph2 architecture is distilled into the MS-DPT student network. Experiments were conducted on two image resolution settings and three self-supervision types. Res indicates the image resolution. M, S, and MS indicate monocular, stereo, and monocular-stereo supervision types, respectively. For error metrics (red), the lower is the better, and for accuracy metrics (blue), the higher is the better.

Significant values are in bold and italics.

### Experimental results on the Make3D dataset

This section presents experimental results on the Make3D dataset to demonstrate the generalization performance of the proposed method. The Make3D dataset contains 400 training images and 134 test samples. The dataset consists of high-resolution RGB images with the size of 2272$\times$1704, and relatively low-resolution ground truth depth maps sized at 55$\times$305. Previous work employed the Make3D dataset to evaluate the zero-shot cross-dataset performance of depth estimation networks^15, 16^. Depth estimation networks trained on the KITTI dataset were evaluated on the Make3D dataset to analyze the generalization performance of the deep learning models.

**Figure 6:**
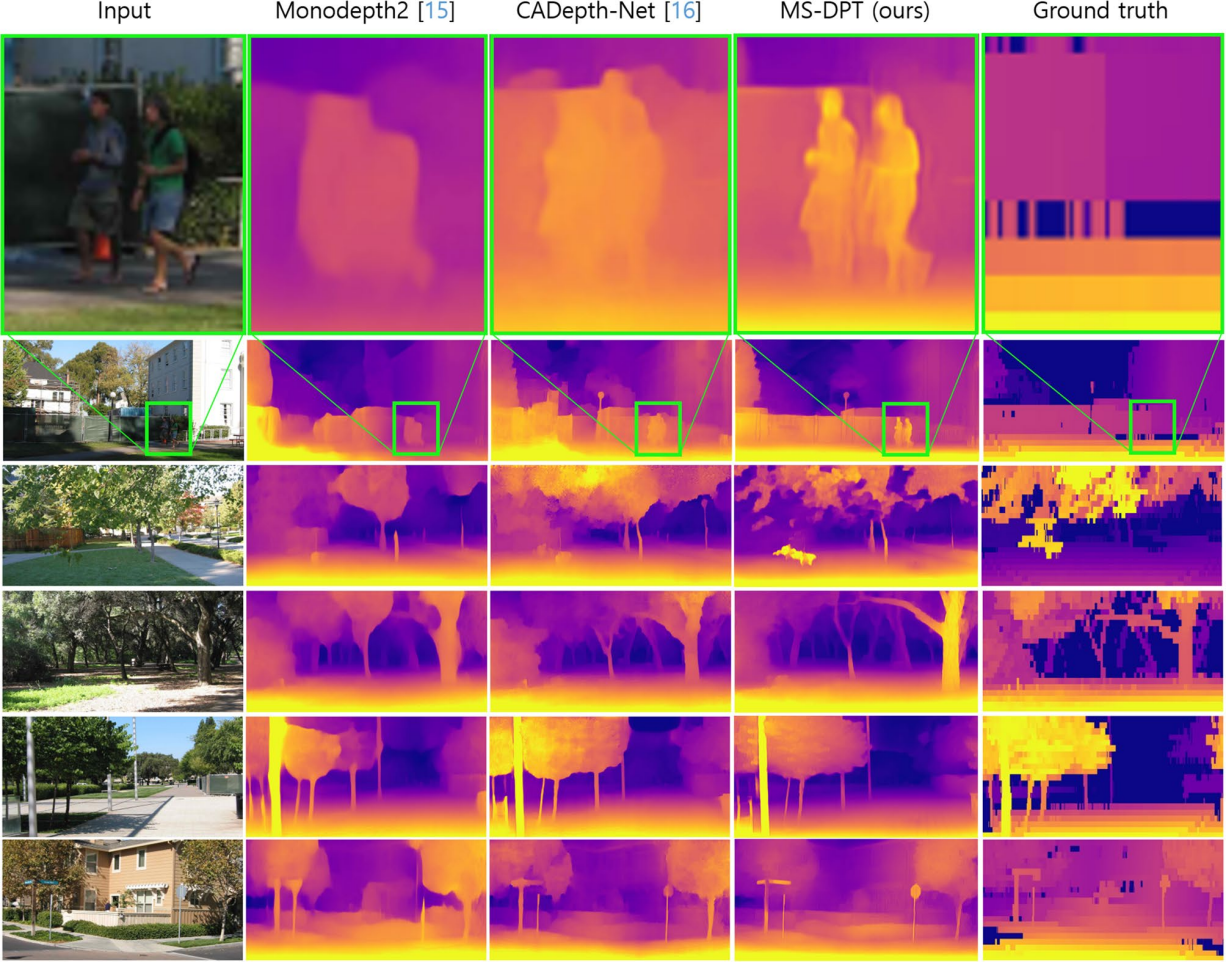
Qualitative comparison with other mehtods on the Make3D dataset^11^. All self-supervised manners were trained on the KITTI dataset^10^.

We evaluated the proposed student model which was trained on the KITTI dataset. This process involved median scaling and 2$\times$1 center crop, following the evaluation protocol in Monodepth2^15^ and CADepth-Net^16^. Table 5 presents the zero-shot cross-dataset performance of our proposed method. The MS-DPT model outperformed all previous methods in all performance metrics. The AbsRel error metric of the MS-DPT model significantly decreased from 0.312 to 0.238 compared to the CADepth-Net model. To demonstrate the significance of the proposed model, we further evaluated the performance of the MS-DPT architecture in a supervised manner. The MS-DPT model was trained on the Make3D dataset, and its performance was compared with previous methods. The error metrics of the supervised model were relatively higher than those observed in the KITTI dataset, due to the insufficient number of training images and small size of ground truth depth maps. Figure 6 presents qualitative results to demonstrate the zero-shot cross-dataset performance of self-supervised methods. As shown in Figure 6, ground truth depth maps tend to be inaccurate, especially for small and thin-shaped objects. Our proposed method demonstrated better quality compared to the previous self-supervised methods by accurately inferring depth maps even for small objects.

**Table 5:**
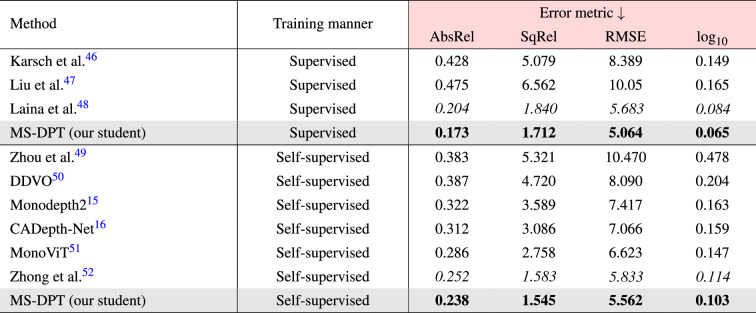
Quantitative comparison with other methods on the Make3D dataset^11^.

All self-supervised methods benefit from median-scaling.

Significant values are in bold and italics.

## Conclusion

This paper proposes a self-supervised depth estimation algorithm based on knowledge distillation. We proposed the MS-DPT model which incorporates multi-scale depth prediction heads onto the previous dense prediction transformer. Moreover, we employed MC-dropout to generate an ensemble of MC estimates for improving the reliability of the teacher network. The student network was trained in a supervised manner based on the multi-scale distillation loss by using depth predictions obtained from the teacher network. To demonstrate the effectiveness of the proposed method, experiments were conducted on the KITTI and Make3D datasets. The proposed method achieved the state-of-the-art performance for self-supervised depth estimation on the KITTI dataset. Furthermore, the proposed method demonstrated better generalization performance on the Make3D dataset compared to previous algorithms, reducing the gap between the performance of supervised and self-supervised methods.

## Data Availability

All data used in this study are available online. The KITTI dataset is available at https://www.cvlibs.net/datasets/kitti/eval_depth.php?benchmark=depth_prediction, and the Make3D dataset is available at http://make3d.cs.cornell.edu/data.html. The code and pretrained model of the proposed method is available at https://github.com/ji-min-song/KD-of-MS-DPT, accessed on 15 March 2023.
